# A community-based approach to indigent selection is difficult to organize in a formal neighbourhood in Ouagadougou, Burkina Faso: a mixed methods exploratory study

**DOI:** 10.1186/1475-9276-13-31

**Published:** 2014-04-16

**Authors:** Valéry Ridde, Clémentine Rossier, Abdramane B Soura, Fiacre Bazié, Kadidiatou Kadio

**Affiliations:** 1 University of Montreal School of Public Health (ESPUM), Montreal, Canada; 2 Research Centre of the University of Montreal Hospital Centre (CRCHUM), Montreal, Canada; 3 Institut Supérieur des Sciences de la Population (ISSP), University of Ouagadougou, Ouagadougou, Burkina Faso; 4 Institut national d’études démographiques, Paris, France

## Abstract

**Background:**

In most African countries, indigents treated at public health centres are supposed to be exempted from user fees. In Africa, most of the available knowledge has to do with targeting processes in rural areas, and little is known about how to select the worst-off in an urban area. In rural communities of Burkina Faso, trials of participatory community-based selection of indigents have been effective. However, the process for selecting indigents in urban areas is not yet clear.

**Methods:**

This study evaluates a community-funded participatory indigent selection process in both a formal (*loti*) and an informal (*non-loti*) neighbourhood in the urban setting of Burkina Faso’s capital. This was an exploratory study to evaluate the processes and effectiveness of participatory targeting. We conducted individual interviews (n = 26) and analyzed secondary qualitative data (eight focus groups, 16 individual interviews). We also used the results of a socioeconomic survey (carried out by the Ouaga HDSS in 2011) of all the households established in the areas, including those of selected indigents.

**Results:**

The coverage of indigent targeting was very low: 0.33% (*loti*) and 0.22% (*non loti*). In the *non loti* neighbourhood, the level of poverty among people selected was higher than the mean level of the poor who were not selected. Some indigents selected in the *loti* neighbourhood were not among the worst-off. The process was difficult to organize in the *loti* neighbourhood; people knew each other less well and were not very available, and there were cases of collusion. The process worked well in the *non loti* neighbourhood.

**Conclusions:**

This intervention research provides new evidence about the feasibility of a community-based selection process in an urban setting in Africa by comparing two different urban settings. The participatory community-based selection process appeared to be suitable for the *non loti* neighbourhood, but other targeting strategies need to be found for *loti* areas. Specific budgets need to be allocated to increase the coverage of indigent targeting.

## Abstract (fr)

Additional non-English language abstract: French

Dans la plupart des pays d’Afrique, les indigents doivent être exemptés du paiement des soins dans les centres de santé publics. En Afrique, la plupart des connaissances disponibles concernent les processus de ciblage realisés en milieu rural, mais très peu de données probantes concernent ces processus en milieu urbain. Des expériences de sélection communautaire participative ont été testées avec efficacité en milieu rural au Burkina Faso. Cependant, on ne sait pas encore comment sélectionner les indigents en milieu urbain.

**Méthodes**

L’étude vise à évaluer une sélection communautaire des indigents financée par la communauté dans un quartier loti et un quartier non loti du milieu urbain de la capitale du Burkina Faso. Il s’agit d’une recherche exploratoire d’évaluation des processus et de l’efficacité du ciblage participatif. Des entrevues individuelles (n = 26) et une analyse de données qualitatives secondaires (8 focus groups, 16 entrevues individuelles) ont été réalisées. Nous avons également utilisé une enquête socio-économique réalisée auprès de tous les ménages des quartiers concernés par l’Observatoire de Population de Ouagadougou en 2011, y compris auprès des ménages des indigents sélectionnés.

**Résultats**

La couverture du ciblage des indigents est très faible, soit 0,33% (loti) et 0,22% (non loti). Les personnes sélectionnées dans le quartier non loti sont plus pauvres que la moyenne des pauvres non sélectionnés. Une partie des indigents sélectionnés dans le quartier loti ne sont pas parmi les plus pauvres. Le processus a été difficile à organiser dans le quartier loti, où les personnes se connaissent moins, sont peu disponibles, et des cas de collusion ont été rencontrés. Le processus s’est bien déroulé en quartier non loti.

Cette recherche interventionnelle génère de nouvelles connaissances sur la faisabilité d’un processus participatif de ciblage en milieu urbain en Afrique en comparant deux milieux. Le processus communautaire participatif semble adapté au quartier non loti en milieu urbain mais d’autres solutions de ciblage doivent être trouvées en quartier loti. Des budgets spécifiques doivent être alloués pour augmenter la couverture du ciblage des indigents.

## Background

In February 2013, the Ministerial Meeting on Universal Health Coverage organized by WHO again raised the issue of targeting public policies. Faced with the predicament of having only limited resources available to improve coverage, some countries, such as Morocco and Nepal, proposed to begin by targeting a few specific populations [1].

Universal coverage has no meaning if it does not provide for the worst-off, i.e., those who are unable to pay user fees imposed at the point of service [2]. However, the issues involved in the targeting of interventions aimed at improving the lot of the worst-off are many and have been debated at length, not only in terms of political economics [3-5] but also with regard to their social and even purely technical aspects [6-8]. In Africa, in the healthcare field, targeting is a key issue, given that most countries have formulated systems of exemptions for the indigent. In fact, most countries charge point-of-service user fees, thereby excluding indigents from access to services [9]. Exemption policies instituted in Africa in the 1990s for the indigent actually benefited others more than the indigent [10]. This raises the question of how indigents should be selected for exemption from user fees, in order to move toward the universal health coverage called for by the World Health Assembly.

Up to now, the debates in the literature have been more theoretical and conceptual than practical. In fact, there have been very few interventions attempted whose evaluation could provide lessons for improvement [11]. The scarcity of documented interventions is especially glaring in Africa [12]. A literature review in the early 2000s showed that, of 122 interventions implemented in 48 countries, only 13 were in sub-Saharan Africa [6].

This lack of evaluated experiences is all the more surprising given the absence of consensus on the most appropriate targeting method (categorical, geographic, individual, etc.) and the most suitable process (community, administrative, etc.). Most authors emphasize the importance of adapting the targeting to the specific context in which it is implemented [6] but “*few studies document the critical ‘how and why’ issues*” [p. 152] [8].

On top of this, in sub-Saharan Africa in particular, there have been almost no experiences of targeting with regard to healthcare user fee exemptions in urban settings. For instance, in Burkina Faso, the few experiences of targeting in urban settings have been published in the grey literature and have not been in the health field, but rather in nutrition and food distribution [13,14]. Most of the available literature for targeting in healthcare concerns rural settings [15-19]. This rural orientation makes sense, since on this continent poor populations are largely concentrated in rural areas. However, the continent is undergoing rapid urbanization, and poverty in African cities is growing. For example, the incidence of poverty in Burkina Faso between 2003 and 2009 remained stable in rural areas (52.3% vs. 52.8%), but increased in urban areas (from 19.9% to 25.2%) [20]. One targeting experiment in an urban neighbourhood of the capital of Mauritania incorporated the user fee exemption into a health mutual. Community selection of indigents was unsuccessful, obliging the program’s initiators subsequently to adopt a more administrative process managed by a specialist [21]. In a user fee exemption system for indigents tested in Madagascar, the targeting was more effective in urban than in rural areas, but the impacts on patients were very limited in both cases [18]. In Ghana, an experiment comparing several processes concluded that in urban settings, where poverty incidence is low, it would be preferable to use means testing (i.e., measuring individuals’ resources), whereas a participatory community-based approach would be more efficient in rural settings [22]. Indeed, given the “*anonymity that leads to a certain social atomization*” [authors’ translation] [23] in African cities, participatory community-based approaches for targeting indigents can be difficult to implement [6]. Given the scarcity of experiences of community-based indigent targeting in African cities, and our desire to find out whether these hypotheses about the difficulty of conducting community-based processes in cities were well-founded, we conducted an experiment in Ouagadougou, the capital city of Burkina Faso. In the following section we present the intervention we tested, before describing the methodology used to evaluate it and the results obtained.

### Intervention

#### General context

The intervention was part of an evidence-production process geared to Burkina Faso’s context. In this country, it has been estimated that 46.7% of the population lives below the poverty threshold and 9% below the extreme poverty threshold [24]. Since the early 1990s, the user fee system has been generalized to all the country’s health centres. All patients must pay for consultations and drugs at the point of service. In public dispensaries, the cost of a consultation is 0.20 USD, which corresponds to the price of a plate of rice or beans in popular restaurants. Added to the consultation cost, there is also the cost of drugs. In rural settings, the average cost of a medical act for a child under five years (including fees, medications, and other treatments provided during the episode of care) is estimated to be around 6 USD [25]. The system for exempting indigents, provided for in official documentation since 1992 and in recent national health policies, have never actually been implemented [26,27]. In fact, fewer than half of sick children go to a health centre, and 99% of the population is not covered by any health insurance system [24]. Policies subsidizing healthcare costs and experiments with exemptions have shown that the financial barrier is one of the main reasons for low utilization of public healthcare services, even when other explanatory factors are involved [25,28]. All population categories are affected by this financial barrier, but the worst-off are the primary victims [29].

Several community-based targeting exercises to exempt the worst-off from user fees had recently been tested in rural areas of Burkina Faso [15,16]. However, to support the authorities, who had decided in their recent national social protection policy to exempt the country’s indigent population from user fees [30], there was also a need for scientific evidence specific to urban areas that could help stimulate a possible national scale-up of local experiences, which up to then had been largely focused in rural areas [31]. Thus, a participatory targeting process adapted to the urban setting and designed according to lessons drawn from a rural experience [15] was implemented in 2011–2012 in Ouagadougou, the capital of Burkina Faso.

### Specific context

To foster evaluation, the intervention was implemented on part of the territory covered by the Ouagadougou Health and Demographic Surveillance System (Ouaga HDSS) [32]. Two of the authors are involved in managing the HDSS, as site leader (AS) and associate site leader (CR). The area covered by the Ouaga HDSS involves five neighbourhoods located at the city’s northern limit. The population monitored by the Ouaga HDSS consists of 81,717 individuals living in 18,310 households in 2012, spread over two neighbourhoods that are serviced, or *loti* (Kilwin, Tanghin), and three neighbourhoods of squatter, or *non loti*, settlements (Nonghin, Polesgo, and Nioko 2). The latter are generally lacking in public sanitation facilities, potable water supply, schools, or public health services. At this time, more than 30% of Ouagadougou’s population lives in *non loti* neighbourhoods [33]. According to a poverty indicator calculated based on households’ goods in the year 2009, it has been estimated that 27.2% of people residing in the *loti* neighbourhoods covered by the Ouaga HDSS are poor, compared with 65.6% in the *non loti* neighbourhoods covered by the Ouaga HDSS [34].

The experiment was conducted in two neighbourhoods representing the most contrasted settings of the Ouaga HDSS territory: Kilwin (a relatively new *loti* neighbourhood, urban, whose level of wealth is comparable to the average for Ouagadougou) and Polesgo (a village attached to the city through the encroachment of *non loti* settlements in the 2000s, whose level of poverty is comparable to that of the rural population as a whole). The more recently the *loti* has been established, the less people know each other and can fairly assess the situations of the others in their neighborhood. Over time, as the *loti* becomes older, people become more familiar with each other. In the *loti* parts of the city, residents are physically separated by the size of their land, while in the *non loti* areas, houses are closer to each other and communication much easier.

Our methodological decision to evaluate two contrasted cases allowed us to compare the interventions in terms of their contexts [35]. Each neighbourhood was linked to a health and social promotion centre (CSPS), which is a primary care centre in the public healthcare system; the two CSPSs were in different health districts. The Ouaga HDSS portion of Kilwin represented about one-third of the inhabitants covered by the CSPS of sector 21, and the Ouaga HDSS portion of Polesgo represented about one-quarter of the inhabitants covered by Polesgo’s CSPS.

### Description of the intervention

The aim of the intervention was to establish committees of volunteers to select indigents in these two neighbourhoods who would then be given cards exempting them from user fees in the CSPS of their health area. In Polesgo, the selection of committee members was done by key informants from the Ouaga HDSS, who were people appointed by the Ouaga HDSS to look after community relations and who knew the neighbourhood well. Because the Ouaga HDSS had not been able to set up a similar system in the *loti* neighbourhoods, the controllers of the Ouaga HDSS field survey team in Kilwin appointed delegates to the committees, who then selected their own members.

Each committee was mandated to select the people they considered to be indigent. They were not given selection criteria; they were expected to base their selection on their own definition of indigence that they would produce through a participatory and consensus-driven process similar to one conducted in a Burkinabè rural setting, which defined an indigent person as “someone who is extremely disadvantaged socially and economically, unable to look after himself (herself) and devoid of internal or external resources” [15]. Each committee submitted the list of persons it considered indigent to the COGES (management committee) of its CSPS for approval, since the COGES is the ultimate source of funding. In effect, the funds used to sustain the exemption come from the CSPS’s own revenues generated by profits from sales to users of drugs and medical acts. These funds are managed by the COGES, which therefore has final say on the list of indigents retained. This use of the funds for indigent exemptions has been enshrined in the national policy since the 1990s [36]. A national directive also reminded the CSPSs that they could plan for this exemption by setting aside a maximum of $400 per year to cover all the indigents in their health area [37]. At the end of the process, the indigents retained by the COGES on the final list were informed of their right to user fee exemptions and were given official cards signed by the Ministry of Social Action, valid for two years.

This entire process, from providing information to all stakeholders at the project’s start, all the way to distributing cards to the selected indigents, took place over a 15-month period between June 2011 and August 2012.

This article presents the findings from evaluating both the processes and the intervention’s effectiveness.

## Methods

### Design

This study involved both a process evaluation and an effectiveness evaluation. In the field of intervention evaluation, process evaluation focuses on understanding how the action works, its strengths and weaknesses, its problems and its challenges [38]. The evaluation of effectiveness, in this article, concentrates on the intervention’s coverage and on targeting errors [39].

### Process analysis

The processes were evaluated using qualitative methods based on primary data collected by the authors (individual interviews, informal interviews, and participant observations) and secondary data from two student theses.

The primary data collected by the authors consisted of individual interviews (n = 26). In the Kilwin neighbourhood, in-depth individual interviews were carried out with indigent persons (three on the final list, and three not retained) and with all delegates to the local selection committees (n = 11). In the Polesgo neighbourhood, in-depth individual interviews were carried out with indigent persons (three on the final list, and three not retained) and all the delegates of the local selection committees (n = 3). As mentioned, the Kilwin neighbourhood is four times greater in size than the Polesgo neighbourhood, which explains the smaller number of selection committees in Polesgo and the differences in numbers of stakeholders contacted for the evaluation.

Then, throughout this process in which the authors were involved in discussions, we conducted informal interviews with all the stakeholders (health workers, district managers, all the selected indigents). Participant observations of all interactions over the 15 months of the process provided further data used in this evaluation.

The interviews were most often conducted in French, and some in Moré, the predominant language in Ouagadougou. Interviews were audio-recorded, transcribed, and translated into French. For the 12 indigents, only the recordings were used. All interviews were focused on obtaining people’s views regarding the intervention’s implementation, its problems, its strengths, the adaptation strategies used by the various actors, etc. Subsequently, we performed a thematic content analysis to identify the key aspects of the intervention’s implementation.

Finally, the analysis was also based on secondary data drawn from the master’s theses of two students in the Health and Population Master’s Program at the ISSP (*Institut Supérieur des Sciences de la Population*) in Ouagadougou supervised by one of the authors (CR). As part of their thesis work, which involved studying part of this intervention’s processes in each of the two neighborhoods, the students conducted eight (combined total) focus groups (n = 53 total participants) and 18 individual interviews with key stakeholders [40,41], with each student working on his own in one neighbourhood.

### Analysis of effectiveness

The intervention coverage is the proportion of indigents selected by the committees at both levels in relation to the total population of the reference area. With respect to targeting errors, the intervention aimed to minimize both inclusion biases (persons selected as indigents who are not) and exclusion biases (persons who are indigent but not selected). We had no data on the incomes or expenses of the households followed by the Ouaga HDSS. However, by comparing the individual characteristics and socioeconomic profiles of the households in which the selected indigents lived against the other households of the Ouaga HDSS that were not selected, we were able to obtain an approximation of the effectiveness of targeting. The hypothesis we wished to test was that the indigents retained by the COGESs lived in more vulnerable households than did the other poor inhabitants of the reference area who were not selected. The data were drawn from the socioeconomic survey of all households carried out by the Ouaga HDSS in 2011 covering the entire population of that area. We used a poverty indicator based on household goods. Households were considered “poor” when they did not have a motorbike, an automobile, a television, or a refrigerator; the methodological details are presented elsewhere [32,42]. Inter-group comparisons of means and ratios were done using t-tests.

We should point out that we did not analyze the effectiveness of targeting with respect to the indigents identified by the local selection committees and then not retained by the COGES (n = 1,054). Pairing the data for each indigent entered in the local committees’ notebooks with the data in the Ouaga HDSS database would have required visiting each of the homes involved, a relatively long process which would have exceeded the budget for this evaluation. Moreover, our objective was to assess the effectiveness of the targeting at the end of the process. The selection made by the local committees is considered here as an intermediate result.

The study received approval from the health ethics committees in Burkina Faso and at the University of Montreal.

## Results

First we present the level of coverage of the intervention, followed by the effectiveness of the targeting, which will then be helpful in understanding the analysis of the processes.

### Very low coverage

Table 1 presents the coverage of the targeting in the two neighbourhoods. The number of indigents retained by the COGESs was very low and similar in both neighbourhoods, at around three indigents per 1,000 inhabitants. While both COGESs followed the same rule dictated by national directives (spend a maximum of $400 per year for indigents), they also selected the number of indigents according to a general (and conservative) assessment of how much revenue their CSPS generated. As one COGES member explained: “Because we know … how much money the COGES has, that’s why our list is shorter”. The numbers of indigents pre-selected by the local selection committees, however, varied considerably. The qualitative data showed that the two neighbourhoods interpreted the definition of indigence differently at the start (see above).

**Table 1:** Coverage of indigent selection in the two sectors of Ouagadougou

	**Kilwin**	**Polesgo**
Type of neighbourhood	*loti*	*non loti*
Total number of inhabitants (2011)	22,418	5,994
Number of local selection committees	11	3
Number of indigents retained by local committees	1 117	25
Coverage of targeting by local committees	5%	0.4%
Number of COGESs	1	1
Number of indigents retained by COGESs	75	13
Coverage of targeting by COGESs	0.33%	0.22%

In Kilwin, while some community selection volunteers retained the definition that had been provided for indigence, others defined it more broadly, using as selection criteria the inability to look after oneself when ill, food insecurity, or inadequate housing conditions. “*When you see someone and you know things are not going well for him, you know [he’s indigent], the person doesn’t even have a good place to sleep, has nothing to eat, when you look at the person’s yard, you can sense it*” (delegate, committee 8, Kilwin). Thus, in this formal neighbourhood, where the standard of living was comparable to the average in Ouagadougou, the definition applied was focused on basic needs.

In Polesgo, where the vast majority of inhabitants lived below the poverty line, as in rural areas, it was not feasible to distinguish indigents from others on the basis of unmet basic needs, given that most of the inhabitants were in that situation. The volunteers thus focused on the individual’s physical incapacity to generate income (disability, advanced age) combined with a total absence of family support. “*We look at people who are disabled and have no material or financial support at all…. They can’t work, and there is absolutely no way they can look after themselves when they are ill because they have nothing at all and no one to help them*” (delegate, committee 1, Polesgo).

The very selective nature of the Polesgo neighbourhood volunteers’ selection process was confirmed by their later reflection in hindsight: *“There were indigents we didn’t include on the list simply because we considered their situation to be less serious than those of the others we selected, but today we see that we should have included them as well, because some of these people are really suffering”* (delegate, committee 2, Polesgo).

### Differences in effectiveness between neighbourhoods

Of the 88 indigents selected by both the local committees and the COGESs, 65 (78.3%) were included in the analysis of effectiveness. This attrition in numbers was related to the mobility of this unstable urban population, and in one case, to death. Individuals (and households) are only entered into the Ouaga HDSS database, by definition, after six months of residence, whereas not all indigents stay put for six months; indeed, some selected indigents left the sector before receiving their card. Moreover, information on household goods was only available for households that the Ouaga HDSS was able to survey when it passed through in 2011 (91.3% of households), which also reduced the number of indigents included in the effectiveness analysis.

Table 2 shows that the level of poverty among people selected in Polesgo was higher than the mean level of the poor who were not selected. The indigents had none of the previously mentioned goods (motorbike, automobile, television, refrigerator). Their households also had fewer mobile phones (63%) than did other poor households (88%), but slightly more bicycles (100% vs. 91.6%). The indigents selected in Polesgo were more often elderly, more often female, and lived more often in households headed by women. They were rarely engaged in economic activity (17% vs. 66% for all the poor), and when they were, it was always in the informal sector (such as gathering and selling sand). The selected indigents were all uneducated, compared with 65% of persons classified as “poor” in Polesgo. The indigents’ characteristics were a function of the definition of indigence used; indeed, it is most often the elderly, women, and people with no education who are afflicted by incapacitating disabilities or social isolation.

**Table 2:** Selected indigents’ characteristics compared with those of the non-selected poor

	**Kilwin ( *loti *)**			**Polesgo ( *non loti *)**		
	**Selected indigents (n = 57)**	**Non-selected poor (n = 1423)**	**p-value**	**Selected indigents (n = 8)**	**Non-selected poor (n = 1390)**	**p-value**
**Individual characteristics**						
Mean age	58.2 (55.0–61.4)	36.1 (35.4–36.8)	0	48.9 (30.8–66.9)	36.5 (35.8–37.2)	0.01
% of people aged 60 years and older	40.4 (27.6–53.1)	7.4 (6.1–8.8)	0	12.5 (0.0–40.2)	7.3 (5.9–8.6)	0.57
% of females	49.1 (36.1–62.1)	51.4 (48.8–54.0)	0.73	62.5 (22.0–100)	48.6 (46.0–51.3)	0.43
% people without education	82.1 (72.1–92.2)	50.0 (50.4–55.6)	0	100	64.8 (62.3–67.3)	0.07
% of people without activity	53.6 (40.0–66.6)	38.3 (35.8–40.9)	0.02	83.3 (52.2–100)	33.5 (31.0–36.0)	0.01
% of people engaged in the informal sector	39.3 (26.5–52.1)	40.3 (37.7–42.8)	0.88	16.7 (0.0–47.9)	43.9 (41.3–46.5)	0.18
% of people employed in the private sector	7.1 (0.4–13.9)	15.1 (13.2–17.0)	0.1	0	20.0 (17.9–22.1)	0.22
**Household characteristics**						
% of households with a bicycle	40.3 (27.0–53.1)	83.2 (81.2–85.1)	0	100	91.6 (90.2–93.1)	0.39
% of households with a motorbike	50.9 (37.9–63.8)	0	0	0	0	
% of households with a television set	21.0 (10.5–31.6)	0	0	0	0	
% of households with a refrigerator	1.8 (1.6–5.2)	0	0	0	0	
% of households with a phone (mobile)	91.2 (83.9–98.6)	91.6 (90.2–93.1)	0.91	62.5 (22.0–100)	87.6 (85.8–89.3)	0.03
% of households with mud-brick houses	35.1 (22.7–47.5)	30.8 (28.4–33.2)	0.49	100	97.9 (97.2–98.7)	0.68
% of households headed by women	29.8 (17.9–41.7)	24.2 (22.0–26.5)	0.34	37.5 (0.0–78.0)	16.3 (14.4–18.3)	0.11

In Kilwin, compared with the poor as a whole in that neighbourhood, the selected indigents were also more often elderly, without economic activity (54% vs. 38%), and uneducated (82% vs. 50%). However, women were not overly represented among the indigents, and the selected indigents had noticeably more goods than the poor as a whole in that sector: 51% of the selected indigents’ households had a motorbike, 21% had a television, and 2% had a refrigerator, while the poor households had none of these items. The selected indigents lived in households that had just as many mobile phones as did the other poor households.

### The role of social relationships in the selection process

Contrary to Polesgo (*non loti* neighbourhood), a portion of the indigents selected in Kilwin (*loti* neighbourhood) were thus not among the worst-off in the sector, if we consider goods owned by the households. These results can have several explanations.

First, the names submitted to the COGES in Kilwin were not listed in any order of degree of indigence. As we saw earlier, the volunteers in Kilwin (contrary to those in Polesgo) interpreted the definition of indigence more variably, resulting in a certain heterogeneity in the indigents selected. The COGES had no way of making any informed choices among these different categories of indigents and therefore did not systematically retain the worst-off in the proposed lists.

Then, in Kilwin, some of the local committees admitted they sometimes made selections based on incomplete information. *“Very often there were people [about whom] we couldn’t get certain information, particularly whether there were any other people who helped them”* (delegate, committee 8, Kilwin). In other local committees, members did not actually know the inhabitants of their own neighbourhood, and so were unable to produce a list of indigents through mutual consultation, as they were supposed to do. Contravening the rules set out at the start of the intervention, some committees roamed across their sector, asking questions in every household. Alerted by the operation, some residents biased their responses. *“There were some people who might tell us they had no help, when that wasn’t the case. Since we listed people based on what they told us, I can’t say with certainty that all the people we selected were actually people who had no assistance”* (delegate, committee 7, Kilwin). On top of that, the members of the COGES who were charged with finalizing the list of indigents also hardly knew the inhabitants of their neighbourhood, which was very large. *“Since we don’t know all these people, we asked [a selection committee] to give us a shorter list with only ten people, since they’re the ones who know each other, and we were overwhelmed”* (COGES representative, Kilwin) [40].

In contrast, the local selection committees in Polesgo were made up of people from the village that had been originally settled along the boundary of Ouagadougou city, and which had only recently become integrated into the capital as a result of the gradual advance of *non loti* settlements over several years. Even though committee members’ knowledge about the newer residents was not perfect, it was still quite good. *“I was born in this sector and have been here ever since. I’m a native of this place and now I’m 32 years old. Because of this, I think I know this sector pretty well, even if there are always new people arriving in the non loti area. The other members … I’ve known them all a long time because we live in this sector together, they know the area as well as I do”* (delegate, committee 1, Polesgo).

In Kilwin, aside from problems related to people being unknown to members, the operation was also tainted by several cases of collusion. Thus, we noted, for instance, that sometimes indigents were all concentrated in the immediate neighbourhood of a delegate to the local selection committee. Elsewhere, committee members themselves and their families were placed on the lists of indigents. Members of committees in this neighbourhood also reported being under great pressure to put certain people on the lists—pressure that was no doubt exacerbated by the lack of discretion that characterized the operation. Thus, in some committees, selection was a matter of accommodation to avoid tension with their populations, as illustrated by this case experienced by a delegate:

*“There was a woman who approached another member of the committee to demand to be put on the list, and my colleague came to me to explain, so I told him to put her on the list, and if she was retained, that would be her luck as well. If not, she was a retired civil servant and her husband also was a retired civil servant, but he had recently passed away, and she continued to receive both pensions. With all that, she wanted to be on the list, and because we didn’t want any trouble, we did it”* (delegate, committee 11, Kilwin).

The way in which these neighbourhood committees were set up no doubt helped facilitate the emergence of such cases of collusion. In Kilwin, it was difficult to identify volunteers to serve on the local selection committees, just as these volunteers then found it difficult to identify indigents. Thus, it was decided to identify only the delegates to each committee, and to ask them to assemble their own committee of volunteers. Sometimes the delegate was the only one who was active.

In Polesgo, on the other hand, the members of the local selection committees were all designated by Ouaga HDSS community liaison agents. Because of this, the members themselves exercised a certain amount of control over their committee’s functioning. These members had all been designated by a third party (the liaison agent), they were active, and they were able to contradict each other. Thus, in Polesgo, some names were eliminated from the proposed list of indigents during internal committee deliberations because members felt they were questionable. As well, in Polesgo the COGES provided a further level of control; for instance, the nurse reported having crossed a suspected case of collusion off the list submitted by the committees [41].

## Discussion

### Methodological limitations

First, we must point out the modest nature of this enterprise, which should be understood as an exploratory study—to our knowledge, the first in Burkina Faso’s capital. This limited scope is especially true given that our study is based only on two case studies. To ensure the viability and sustainability of the experiment, the funds invested for indigent care were consistent with local directives. In fact, we respected the national directives for indigent coverage, which stipulate that CSPSs must spend no more than $400 per year on user fee exemptions for indigents in their health area [37]. Thus, this experiment is primarily useful for analyzing the process implemented, and less so for assessing its effectiveness. The very low number of indigents retained and the decision not to seek out the personal information of those selected by the local committees (1,054 not selected in the end by the COGES) limit the significance of any potential statistical analyses. It should be noted, however, that the methodological challenges associated with evaluating targeting in Burkina Faso’s capital are not new; other researchers have encountered the same challenges when trying to obtain consumption data or to find households for an evaluation [43].

### Confirmation of conflicts of interest and of the need for State funding

This study confirmed the existence of conflict of interest, as the very low coverage of indigents who should have received the user fee exemption funded by the COGESs was due, on one hand, to the low amount officially allotted to this purpose ($400 per CSPS) and, on the other, to the fact that this amount was intended to be funded from the profits generated by the local sale of drugs and medical consultations to CSPS users. In effect, while the desire to make the indigent exemption sustainable is laudable, it is nevertheless hampered by the meagre budget allotment ($400) and made impossible by the limited contributive capacity of households, the low utilization of health services, and the absence of any pooling of resources at the national level. In trials conducted in Africa in which the funding was also endogenous and local, the same challenge of very low coverage was observed, always under 1% [18,21,39]. On the other hand, when communities are not in conflict of interest and the exemption is funded by an external source that is not local, coverage can approach the level of the needs of the worst-off, as has been shown in Burkina Faso and Cambodia [16,44]. In Burkina Faso, a study showed that, given the low utilization of CSPSs because of user fees, the resources generated locally were only able to support free services for less than 1% of the population [45]. In such a context, any attempts to broaden the targeted coverage will of necessity require supplementary funding from the State or partner funding agencies. This is, in fact, precisely the experiment that the government will undertake in 2014 with World Bank support. As part of its plan to implement results-based funding in 12 districts of the country, more than 200 CSPSs will reproduce the community-based targeting process. However, the funding will be provided by the World Bank, and the communities will be able to select up to 20% of their population to be offered user fees exemptions. While this project will not resolve the matter of the exemption’s sustainability, it will help produce evidence on its feasibility and its effectiveness at a larger scale and with a much broader targeted coverage. However, the project will not be implemented in the capital, but only in rural areas, where the process carried out in medium-sized cities should nevertheless contribute to developing knowledge on targeting in urban areas in Africa.

### The challenge of finding a process suited to urban social settings

In urban settings, community-based targeting is rarely used because “*close neighbours may not know each other well and boundaries between ‘communities’ may be very blurry”* (p. 61) [6]. However, this level of mutual knowledge may vary from one location to another, including in a capital city such as Ouagadougou, where we saw that there were two types (to put it simply) of social organization. The community-based social control that characterized Polesgo made it possible to respect the targeting rules and avoid the risks of collusion. This community-based social control is typical of a village social structure, which in this case was reproduced in the city; the results were exactly like those observed in trials conducted elsewhere in rural areas of Burkina Faso [15]. Polesgo was in fact a village on the periphery of Ouagadougou which became an informal neighbourhood of the capital in the mid-2000s. Nearly one-fifth of the working population in the part of that neighbourhood followed by the Ouaga HDSS is still working in agriculture today [46]. However, the results obtained in Polesgo’s indigent selection process cannot be generalized to all the other *non loti* urban areas in Burkina Faso without further verification. Further studies could be carried out to test other participatory processes in *non loti* areas different from the one in this study. The project funded by the World Bank targeting 20% of the population in several rural areas, which include medium-sized cities, should produce such knowledge.

In the *loti* neighbourhood of Kilwin, we saw how the absence of a closely woven social fabric on a mesoscopic scale (since this fabric does exist at the microscopic level, such as a street, or religious community, or professional groups) could partly explain the difficulties in implementing the indigent selection process. However, we should also point out that the low level of mutual acquaintanceship in the neighbourhood was not the only impediment to community-based targeting in an urban setting. In fact, in both neighbourhoods it was difficult to mobilize volunteers, something that was accomplished more easily in rural settings in Burkina Faso [15,16]. The costs incurred (compensation of various expenses) were much higher than in rural settings, and the process dragged on due to low motivation among the people involved (it took 15 months to complete the intervention). It was also in Kilwin that the committees did not fully respect the rules set out for the implementation of the intervention. Failure to maintain fidelity of implementation is not in itself a bad thing, as an intervention will occasionally require innovation to be effective [47]; however, that was not the case in this study.

This difference between the two neighbourhoods confirmed the previous experiences of the Ouaga HDSS. For example, it had never been possible to establish community liaison agents in the *loti* neighbourhoods followed by the Ouaga HDSS, despite efforts expended over more than a year in 2009–2010. In contrast to the *non loti* neighbourhoods, the liaison agents appointed in the *loti* neighbourhoods were not interested in work offering low wages. It also appeared that they did not relay information effectively to the community, contrary to what was done in the *non loti* neighbourhoods. This showed that there were real local communities in the *non loti* neighbourhoods followed (where the prospect of becoming *loti* acted as a strong informal integrative force), unlike in the city’s *loti* neighbourhoods (where interactions between neighbours were strong but generally limited to a block of houses, and where people formed groups based on social affinities, such as churches, that did not coincide with the neighbourhood’s boundaries).

This research also shows the need for continued testing and study of targeting processes (community-based, proxy means testing, etc.) in different settings. In fact, scaling up a community-based process to the entire country does not appear to be realistic, any more than would be using a single method of targeting. Solutions need to be found that can be tailored to different contexts and are cost-effective [6].

## Conclusions

To our knowledge, this is the first intervention research on targeting of the worst-off to be organized in an urban environment in Burkina Faso in the health field in order to improve indigents’ access to care. Moreover, it provides new knowledge for the rest of Africa, as experiences with targeting in urban areas are rare in sub-Saharan Africa. The study’s originality also lies in the adoption of a non-monolithic approach to study the urban setting, by taking into account both formal and informal environments that currently characterize Africa’s urban development. Thus, in the current context of massive development of cities and of populations living in urban areas in Africa, this study will inform the general debates both on targeting in urban areas and, more broadly, on universal healthcare coverage [48], where access to care for indigents is imperative.

More locally, the results of this study in the capital, as well as of those undertaken in rural settings, could be helpful to the State in implementing three recent policies that include provisions for the care of indigents. However, for such policies to work, processes clearly need to be better adapted to different contexts (urban/rural) and the State must provide additional funding to avoid the conflicts of interest mentioned earlier. First, the national social protection policy formulated in 2012 announced the State’s intention of exempting indigents from user fees subsequent to, among other things, a community-based selection process such as the one studied in this article [30]. Then there is universal health insurance, currently being planned and expected to be enacted into law in 2013, which will include provisions to fund indigent care. Finally, the State-funded childbirth subsidy policy that provides for user fee exemptions for indigent women remains to be properly implemented [49,50].

It is therefore becoming essential, now, to organize the processes for identifying indigents. The idea has already been advanced elsewhere that the community-based participatory process could definitely be scaled up nationally in rural areas [31,51]. In urban settings, however, our exploratory study showed that this community-based participatory process appears not to be the most suitable in *loti* neighbourhoods. Other community-based approaches could be explored in urban settings. However, in cities, unlike in rural settings, professionals from the Ministry of Social Action (MSA) are present, available, and experts in this matter. They know the criteria that should be used for indigent selection [52]. What they are clearly lacking are the means to carry out their work, as they are most often confined to their services without the resources required to go into those neighbourhoods so that they can identify indigents before the latter present at health centres, which, of course, happens infrequently. However, other studies could also be conducted in urban settings to determine whether local associations and other neighbourhood-based associations might also be mobilized to support these MSA agents.

## Competing interests

The authors declare that they have no competing interests.

## Authors’ contributions

VR, CR, ABS and KK developed the original study and intervention design. CR, ABS and FB piloted the intervention implementation with support from KK and VR. CR and AR were responsible for data collection. CR and ABS conducted the data analysis, which was subsequently reviewed with VR and KK. VR wrote the manuscript with contributions from all authors. All authors approved the final manuscript.
